# (*E*)-Methyl 2-({2-eth­oxy-6-[(*E*)-(hy­droxy­imino)­meth­yl]phen­oxy}meth­yl)-3-phenyl­acrylate

**DOI:** 10.1107/S1600536812014596

**Published:** 2012-04-13

**Authors:** E. Govindan, G. Ganesh, J. Srinivasan, M. Bakthadoss, A. SubbiahPandi

**Affiliations:** a Department of Physics, Presidency College (Autonomous), Chennai 600 005, India; b Department of Physics, SMK Fomra Institute of Technology, Thaiyur, Chennai 603 103, India; c Department of Organic Chemistry, University of Madras, Guindy Campus, Chennai 600 025, India

## Abstract

In the title compound, C_20_H_21_NO_5_, the dihedral angle between the mean planes through the two rings is 47.1 (8)°. The enoate group assumes an extended conformation. The hy­droxy­ethanimine group is essentially coplanar with the benzene ring, the largest deviation from the mean plane being 0.061 (1) Å for the O atom. In the crystal, mol­ecules are linked into cyclic centrosymmetric dimers with an *R*_2_^2^(6) motif *via* pairs of O—H⋯N hydrogen bonds. Inter­molecular C—H⋯O hydrogen bonds form a *C*(8) chain along the *b* axis. The crystal packing is further stabilized by C—H⋯π inter­actions.

## Related literature

For the biological activity of caffeic acids and their esters, see: Hwang *et al.* (2001 ▶); Altug *et al.* (2008 ▶); Ates *et al.* (2006 ▶); Atik *et al.* (2006 ▶); Chaudhuri (2003 ▶); Padinchare *et al.* (2001 ▶). For a related structure, see: SakthiMurugesan *et al.* (2011 ▶). For graph-set analysis of hydrogen bonds, see: Bernstein *et al.* (1995 ▶).
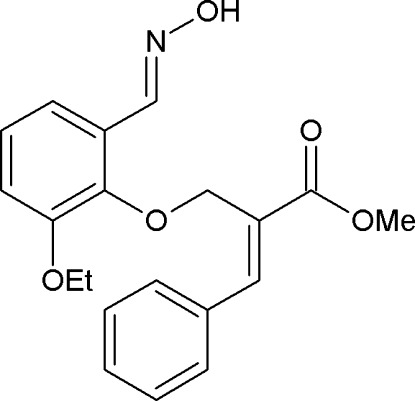


## Experimental

### 

#### Crystal data

C_20_H_21_NO_5_
*M* *_r_* = 355.38
Monoclinic, 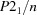

*a* = 7.4009 (3) Å
*b* = 22.1125 (10) Å
*c* = 11.3681 (5) Å
β = 103.561 (1)°
*V* = 1808.55 (14) Å^3^
*Z* = 4
Mo *K*α radiation
μ = 0.09 mm^−1^
*T* = 293 K
0.25 × 0.22 × 0.19 mm

#### Data collection

Bruker APEXII CCD area detector diffractometer
Absorption correction: multi-scan (*SADABS*; Sheldrick, 1996 ▶) *T*_min_ = 0.978, *T*_max_ = 0.983
25247 measured reflections
6042 independent reflections
4293 reflections with *I* > 2σ(*I*)
*R*_int_ = 0.027

#### Refinement

*R*[*F* ^2^ > 2σ(*F* ^2^)] = 0.053
*wR*(*F* ^2^) = 0.167
*S* = 1.02
6042 reflections
238 parameters
H-atom parameters constrained
Δρ_max_ = 0.50 e Å^−3^
Δρ_min_ = −0.24 e Å^−3^

### 

Data collection: *APEX2* (Bruker, 2004 ▶); cell refinement: *SAINT* (Bruker, 2004 ▶); data reduction: *SAINT*; program(s) used to solve structure: *SHELXS97* (Sheldrick, 2008 ▶); program(s) used to refine structure: *SHELXL97* (Sheldrick, 2008 ▶); molecular graphics: *ORTEP-3* (Farrugia, 1997 ▶); software used to prepare material for publication: *SHELXL97* and *PLATON* (Spek, 2009 ▶).

## Supplementary Material

Crystal structure: contains datablock(s) global, I. DOI: 10.1107/S1600536812014596/rn2100sup1.cif

Structure factors: contains datablock(s) I. DOI: 10.1107/S1600536812014596/rn2100Isup2.hkl

Supplementary material file. DOI: 10.1107/S1600536812014596/rn2100Isup3.cml

Additional supplementary materials:  crystallographic information; 3D view; checkCIF report

## Figures and Tables

**Table 1:** Hydrogen-bond geometry (Å, °) *Cg*2 is the centroid of the C15–C20 ring.

*D*—H⋯*A*	*D*—H	H⋯*A*	*D*⋯*A*	*D*—H⋯*A*
O1—H1*A*⋯N1^i^	0.82	2.08	2.8121 (15)	149
C4—H4⋯O4^ii^	0.93	2.59	3.2379 (18)	127
C20—H20⋯O1^iii^	0.93	2.59	3.466 (2)	157
C9—H9*B*⋯*Cg*2^iv^	0.96	2.79	3.616 (2)	145

Symmetry codes: (i) 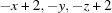; (ii) 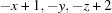; (iii) 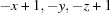; (iv) 
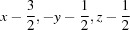
.

**Fig. 1.**
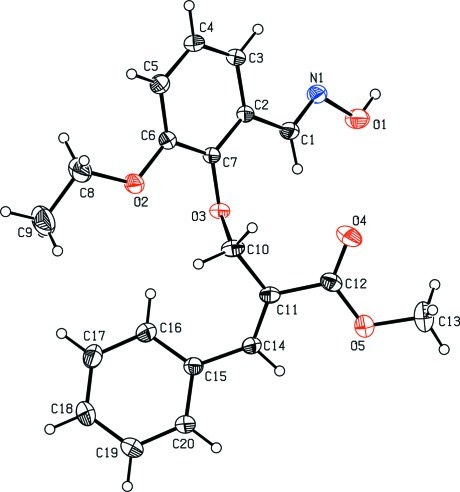
View of the title molecule with the atom labelling scheme. The displacement ellipsoids are drawn at the 30% probability level while the H atoms are shown as small spheres of arbitrary radii.

**Fig. 2.**
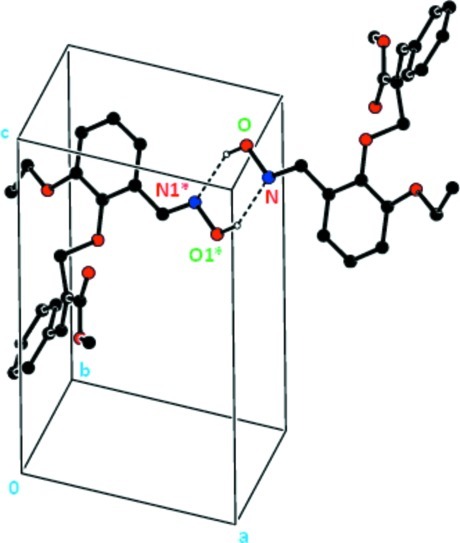
The crystal structure showing the centrosymmetric hydrogen bond motif R22(6). For the sake of clarity, the H atoms not involved in the motif have been omitted. The atoms marked with an asterisk (*) are at the symmetry position (2-x, -y, 2-z). The dashed lines indicate the hydrogen bonds.

## supplementary crystallographic information

### Comment

Some naturally occurring caffeic acids and their esters attract much attention in biology and medicine (Hwang *et al.*, 2001; Altug *et al.*, 2008). These compounds show antiviral, antibacterial, vasoactive, antiatherogenic, antiproliferative, antioxidant and anti-inflammatory properties (Atik *et al.*, 2006; Padinchare *et al.*, 2001; Ates *et al.*, 2006). Oximes are a classical type of chelating ligand which are widely used in coordination and analytical chemistry (Chaudhuri, 2003).

In the title compound (see Fig. 1) the bond lengths and angles agree with those observed in other acrylate derivatives (SakthiMurugesan *et al.*, 2011). The whole molecule is not planar as the dihedral angle between the two phenyl rings is 47.1 (8)°. The oxime C—N has an E configuration. The hydroxyethanimine group is essentially coplanar with the benzene ring, the largest deviation from the mean plane being 0.004 (1) Å for the C2 atom.

The ether group assumes an extended conformation as can be seen from torsion angles C11—C12—O5—C13 [-174.7 (1) °] and C10—C11—C12—O5 [170.7 (1) °]. C—H···O hydrogen bonds (see Table 1) form C(8) chains along (Bernstein *et al.* 1995) the *b* axis. The hydroxyethanimine group in the molecules are linked into cyclic centrosymmetric dimers *via* O—H···N hydrogen bonds with the *R*_2_^2^(6) motif. The closest C—H-centroid separation is 3.6 Å therefore there are not significant C—H···π interactions. In addition to van der Waals interactions the crystal packing is stabilized by C–H···O and O–H···N interactions.

### Experimental

To a stirred solution of (*E*)-methyl 2-((2-ethoxy-6-formylphenoxy)methyl) -3-phenylacrylate (4 mmol) in 10 ml of EtOH/H_2_O mixture (1:1) was added NH_2_OH.HCl (6 mmol) in the presence of 50% NaOH at room temperature. Then the reaction mixture was allowed to stir at room temperature for 1.5 h. After completion of the reaction, solvent was removed and the crude mass was diluted with water (15 ml) and extracted with ethyl acetate (3 *x* 15 ml). The combined organic layer was washed with brine (2 *x* 10 ml) and dried over anhydrous Na_2_SO_4_ and then evaporated under reduced pressure to obtain (*E*)-methyl2-((2-ethoxy- 6-((*E*)-(hydroxyimino)methyl)phenoxy)methyl)-3-phenylacrylate as a colourless solid.

### Refinement

All H atoms were fixed geometrically and allowed to ride on their parent C atoms, with C—H distances fixed in the range 0.93–0.97 Å with *U*_iso_(H) = 1.5*U*_eq_(C) for methyl H 1.2*U*_eq_(C) for other H atoms.

### Crystal data

**Table:**

C_20_H_21_NO_5_	*F*(000) = 752
*M**_r_* = 355.38	*D*_x_ = 1.305 Mg m^−^^3^*D*_m_ = 1.375 Mg m^−^^3^*D*_m_ measured by not measured
Monoclinic, *P*2_1_/*n*	Mo *K*α radiation, λ = 0.71073 Å
Hall symbol: -P 2yn	Cell parameters from 6042 reflections
*a* = 7.4009 (3) Å	θ = 1.8–31.6°
*b* = 22.1125 (10) Å	µ = 0.09 mm^−^^1^
*c* = 11.3681 (5) Å	*T* = 293 K
β = 103.561 (1)°	Block, white crystalline
*V* = 1808.55 (14) Å^3^	0.25 × 0.22 × 0.19 mm
*Z* = 4	

### Data collection

**Table:**

Bruker APEXII CCD area detector diffractometer	6042 independent reflections
Radiation source: fine-focus sealed tube	4293 reflections with *I* > 2σ(*I*)
Graphite monochromator	*R*_int_ = 0.027
ω and φ scans	θ_max_ = 31.6°, θ_min_ = 1.8°
Absorption correction: multi-scan (*SADABS*; Sheldrick, 1996)	*h* = −10→10
*T*_min_ = 0.978, *T*_max_ = 0.983	*k* = −32→32
25247 measured reflections	*l* = −14→16

### Refinement

**Table:**

Refinement on *F*^2^	Primary atom site location: structure-invariant direct methods
Least-squares matrix: full	Secondary atom site location: difference Fourier map
*R*[*F*^2^ > 2σ(*F*^2^)] = 0.053	Hydrogen site location: inferred from neighbouring sites
*wR*(*F*^2^) = 0.167	H-atom parameters constrained
*S* = 1.02	*w* = 1/[σ^2^(*F*_o_^2^) + (0.0873*P*)^2^ + 0.3814*P*] where *P* = (*F*_o_^2^ + 2*F*_c_^2^)/3
6042 reflections	(Δ/σ)_max_ < 0.001
238 parameters	Δρ_max_ = 0.50 e Å^−^^3^
0 restraints	Δρ_min_ = −0.24 e Å^−^^3^

### Special details

**Table:**

Geometry. All e.s.d.'s (except the e.s.d. in the dihedral angle between two l.s. planes) are estimated using the full covariance matrix. The cell e.s.d.'s are taken into account individually in the estimation of e.s.d.'s in distances, angles and torsion angles; correlations between e.s.d.'s in cell parameters are only used when they are defined by crystal symmetry. An approximate (isotropic) treatment of cell e.s.d.'s is used for estimating e.s.d.'s involving l.s. planes.
Refinement. Refinement of *F*^2^ against ALL reflections. The weighted *R*-factor *wR* and goodness of fit *S* are based on *F*^2^, conventional *R*-factors *R* are based on *F*, with *F* set to zero for negative *F*^2^. The threshold expression of *F*^2^ > σ(*F*^2^) is used only for calculating *R*-factors(gt) *etc*. and is not relevant to the choice of reflections for refinement. *R*-factors based on *F*^2^ are statistically about twice as large as those based on *F*, and *R*- factors based on ALL data will be even larger.

### Fractional atomic coordinates and isotropic or equivalent isotropic displacement parameters (Å^2^)

**Table:**

	*x*	*y*	*z*	*U*_iso_*/*U*_eq_	
C1	0.66305 (18)	0.04722 (6)	0.86324 (12)	0.0369 (3)	
H1	0.6348	0.0414	0.7799	0.044*	
C2	0.52766 (17)	0.07679 (6)	0.91943 (11)	0.0313 (2)	
C3	0.5530 (2)	0.08059 (6)	1.04563 (12)	0.0385 (3)	
H3	0.6589	0.0641	1.0960	0.046*	
C4	0.4225 (2)	0.10847 (7)	1.09493 (12)	0.0423 (3)	
H4	0.4392	0.1097	1.1786	0.051*	
C5	0.2661 (2)	0.13482 (7)	1.02182 (13)	0.0415 (3)	
H5	0.1791	0.1539	1.0563	0.050*	
C6	0.23961 (18)	0.13273 (6)	0.89702 (11)	0.0337 (3)	
C7	0.36826 (16)	0.10210 (5)	0.84560 (10)	0.0291 (2)	
C8	−0.0070 (3)	0.20593 (10)	0.86177 (17)	0.0632 (5)	
H8A	0.0740	0.2293	0.9250	0.076*	
H8B	−0.1026	0.1872	0.8951	0.076*	
C9	−0.0933 (3)	0.24597 (10)	0.7579 (2)	0.0772 (6)	
H9A	0.0013	0.2608	0.7202	0.116*	
H9B	−0.1532	0.2795	0.7867	0.116*	
H9C	−0.1834	0.2234	0.7000	0.116*	
C10	0.17647 (18)	0.06976 (7)	0.65716 (11)	0.0372 (3)	
H10A	0.0762	0.0989	0.6349	0.045*	
H10B	0.1401	0.0393	0.7085	0.045*	
C11	0.21243 (17)	0.04080 (6)	0.54594 (11)	0.0329 (3)	
C12	0.28726 (19)	−0.02179 (7)	0.56460 (13)	0.0388 (3)	
C13	0.3504 (3)	−0.11301 (8)	0.4760 (2)	0.0604 (5)	
H13A	0.4669	−0.1161	0.5346	0.091*	
H13B	0.3642	−0.1283	0.3996	0.091*	
H13C	0.2580	−0.1362	0.5028	0.091*	
C14	0.17680 (17)	0.06620 (6)	0.43559 (11)	0.0335 (3)	
H14	0.2015	0.0421	0.3742	0.040*	
C15	0.10419 (17)	0.12684 (6)	0.39869 (11)	0.0335 (3)	
C16	0.1278 (2)	0.17773 (7)	0.47358 (14)	0.0432 (3)	
H16	0.1968	0.1747	0.5531	0.052*	
C17	0.0490 (2)	0.23275 (7)	0.43013 (16)	0.0513 (4)	
H17	0.0651	0.2663	0.4808	0.062*	
C18	−0.0529 (2)	0.23794 (8)	0.31254 (17)	0.0531 (4)	
H18	−0.1072	0.2747	0.2844	0.064*	
C19	−0.0744 (2)	0.18858 (8)	0.23667 (15)	0.0522 (4)	
H19	−0.1420	0.1921	0.1570	0.063*	
C20	0.0045 (2)	0.13382 (7)	0.27890 (13)	0.0422 (3)	
H20	−0.0091	0.1009	0.2266	0.051*	
N1	0.81897 (15)	0.02940 (6)	0.92689 (10)	0.0383 (3)	
O1	0.92841 (16)	0.00226 (6)	0.85611 (10)	0.0534 (3)	
H1A	1.0254	−0.0101	0.9001	0.080*	
O2	0.09792 (15)	0.16043 (5)	0.81645 (9)	0.0468 (3)	
O3	0.34510 (11)	0.09991 (4)	0.72187 (7)	0.0319 (2)	
O4	0.3345 (2)	−0.04452 (6)	0.66304 (12)	0.0700 (4)	
O5	0.29384 (17)	−0.05077 (5)	0.46293 (10)	0.0503 (3)	

### Atomic displacement parameters (Å^2^)

**Table:**

	*U*^11^	*U*^22^	*U*^33^	*U*^12^	*U*^13^	*U*^23^
C1	0.0359 (6)	0.0413 (7)	0.0303 (6)	0.0062 (5)	0.0017 (5)	−0.0023 (5)
C2	0.0332 (5)	0.0295 (6)	0.0281 (5)	0.0018 (4)	0.0011 (4)	−0.0014 (4)
C3	0.0446 (7)	0.0380 (7)	0.0282 (6)	0.0048 (5)	−0.0008 (5)	0.0006 (5)
C4	0.0565 (8)	0.0434 (7)	0.0256 (6)	0.0044 (6)	0.0067 (5)	−0.0007 (5)
C5	0.0496 (8)	0.0433 (7)	0.0336 (7)	0.0087 (6)	0.0140 (6)	−0.0007 (5)
C6	0.0362 (6)	0.0328 (6)	0.0312 (6)	0.0052 (5)	0.0061 (5)	0.0005 (5)
C7	0.0319 (5)	0.0292 (5)	0.0247 (5)	0.0005 (4)	0.0038 (4)	0.0003 (4)
C8	0.0637 (10)	0.0767 (13)	0.0540 (10)	0.0377 (10)	0.0236 (8)	0.0090 (9)
C9	0.0768 (14)	0.0746 (14)	0.0834 (15)	0.0377 (11)	0.0253 (12)	0.0154 (11)
C10	0.0307 (6)	0.0521 (8)	0.0277 (6)	−0.0065 (5)	0.0046 (4)	−0.0026 (5)
C11	0.0293 (5)	0.0399 (6)	0.0274 (6)	−0.0034 (5)	0.0021 (4)	−0.0004 (5)
C12	0.0347 (6)	0.0416 (7)	0.0364 (7)	−0.0033 (5)	0.0007 (5)	0.0059 (5)
C13	0.0545 (9)	0.0386 (8)	0.0902 (14)	0.0061 (7)	0.0213 (9)	0.0016 (8)
C14	0.0353 (6)	0.0363 (6)	0.0275 (6)	0.0001 (5)	0.0044 (5)	−0.0028 (5)
C15	0.0321 (5)	0.0375 (6)	0.0300 (6)	0.0007 (5)	0.0058 (4)	−0.0004 (5)
C16	0.0461 (7)	0.0426 (8)	0.0376 (7)	0.0019 (6)	0.0030 (6)	−0.0063 (6)
C17	0.0570 (9)	0.0411 (8)	0.0550 (9)	0.0062 (7)	0.0117 (7)	−0.0097 (7)
C18	0.0524 (9)	0.0442 (8)	0.0607 (10)	0.0163 (7)	0.0091 (7)	0.0044 (7)
C19	0.0556 (9)	0.0531 (9)	0.0410 (8)	0.0107 (7)	−0.0023 (7)	0.0050 (7)
C20	0.0507 (8)	0.0405 (7)	0.0318 (6)	0.0032 (6)	0.0023 (6)	−0.0018 (5)
N1	0.0344 (5)	0.0451 (6)	0.0338 (5)	0.0073 (4)	0.0043 (4)	−0.0037 (5)
O1	0.0444 (6)	0.0776 (8)	0.0371 (5)	0.0225 (5)	0.0070 (4)	−0.0060 (5)
O2	0.0454 (5)	0.0547 (6)	0.0384 (5)	0.0229 (5)	0.0059 (4)	0.0001 (4)
O3	0.0299 (4)	0.0403 (5)	0.0238 (4)	−0.0013 (3)	0.0032 (3)	0.0005 (3)
O4	0.0975 (11)	0.0573 (8)	0.0452 (7)	0.0093 (7)	−0.0035 (7)	0.0172 (6)
O5	0.0609 (7)	0.0410 (6)	0.0482 (6)	0.0104 (5)	0.0109 (5)	0.0010 (5)

### Geometric parameters (Å, º)

**Table:**

C1—N1	1.2723 (16)	C10—H10B	0.9700
C1—C2	1.4627 (18)	C11—C14	1.3429 (18)
C1—H1	0.9300	C11—C12	1.487 (2)
C2—C7	1.3947 (16)	C12—O4	1.2014 (17)
C2—C3	1.4051 (18)	C12—O5	1.3324 (19)
C3—C4	1.371 (2)	C13—O5	1.436 (2)
C3—H3	0.9300	C13—H13A	0.9600
C4—C5	1.385 (2)	C13—H13B	0.9600
C4—H4	0.9300	C13—H13C	0.9600
C5—C6	1.3866 (19)	C14—C15	1.4687 (18)
C5—H5	0.9300	C14—H14	0.9300
C6—O2	1.3650 (15)	C15—C20	1.3970 (18)
C6—C7	1.4029 (17)	C15—C16	1.3971 (19)
C7—O3	1.3774 (14)	C16—C17	1.389 (2)
C8—O2	1.4382 (19)	C16—H16	0.9300
C8—C9	1.494 (3)	C17—C18	1.377 (2)
C8—H8A	0.9700	C17—H17	0.9300
C8—H8B	0.9700	C18—C19	1.377 (2)
C9—H9A	0.9600	C18—H18	0.9300
C9—H9B	0.9600	C19—C20	1.381 (2)
C9—H9C	0.9600	C19—H19	0.9300
C10—O3	1.4536 (15)	C20—H20	0.9300
C10—C11	1.4957 (18)	N1—O1	1.4034 (15)
C10—H10A	0.9700	O1—H1A	0.8200
			
N1—C1—C2	120.86 (12)	C14—C11—C12	120.50 (12)
N1—C1—H1	119.6	C14—C11—C10	125.14 (13)
C2—C1—H1	119.6	C12—C11—C10	114.32 (11)
C7—C2—C3	118.81 (12)	O4—C12—O5	123.03 (15)
C7—C2—C1	119.08 (11)	O4—C12—C11	122.64 (15)
C3—C2—C1	122.11 (11)	O5—C12—C11	114.32 (12)
C4—C3—C2	120.40 (12)	O5—C13—H13A	109.5
C4—C3—H3	119.8	O5—C13—H13B	109.5
C2—C3—H3	119.8	H13A—C13—H13B	109.5
C3—C4—C5	120.89 (12)	O5—C13—H13C	109.5
C3—C4—H4	119.6	H13A—C13—H13C	109.5
C5—C4—H4	119.6	H13B—C13—H13C	109.5
C4—C5—C6	119.84 (13)	C11—C14—C15	128.85 (12)
C4—C5—H5	120.1	C11—C14—H14	115.6
C6—C5—H5	120.1	C15—C14—H14	115.6
O2—C6—C5	125.00 (12)	C20—C15—C16	117.84 (13)
O2—C6—C7	115.26 (11)	C20—C15—C14	116.97 (12)
C5—C6—C7	119.70 (12)	C16—C15—C14	125.18 (12)
O3—C7—C2	119.05 (11)	C17—C16—C15	120.45 (14)
O3—C7—C6	120.55 (10)	C17—C16—H16	119.8
C2—C7—C6	120.27 (11)	C15—C16—H16	119.8
O2—C8—C9	107.33 (15)	C18—C17—C16	120.46 (15)
O2—C8—H8A	110.2	C18—C17—H17	119.8
C9—C8—H8A	110.2	C16—C17—H17	119.8
O2—C8—H8B	110.2	C17—C18—C19	119.90 (15)
C9—C8—H8B	110.2	C17—C18—H18	120.1
H8A—C8—H8B	108.5	C19—C18—H18	120.1
C8—C9—H9A	109.5	C18—C19—C20	120.01 (15)
C8—C9—H9B	109.5	C18—C19—H19	120.0
H9A—C9—H9B	109.5	C20—C19—H19	120.0
C8—C9—H9C	109.5	C19—C20—C15	121.30 (14)
H9A—C9—H9C	109.5	C19—C20—H20	119.4
H9B—C9—H9C	109.5	C15—C20—H20	119.4
O3—C10—C11	108.81 (10)	C1—N1—O1	112.01 (11)
O3—C10—H10A	109.9	N1—O1—H1A	109.5
C11—C10—H10A	109.9	C6—O2—C8	117.90 (12)
O3—C10—H10B	109.9	C7—O3—C10	114.80 (9)
C11—C10—H10B	109.9	C12—O5—C13	116.09 (14)
H10A—C10—H10B	108.3		
			
N1—C1—C2—C7	172.39 (13)	C12—C11—C14—C15	−179.71 (12)
N1—C1—C2—C3	−7.6 (2)	C10—C11—C14—C15	2.7 (2)
C7—C2—C3—C4	0.3 (2)	C11—C14—C15—C20	−152.45 (14)
C1—C2—C3—C4	−179.74 (14)	C11—C14—C15—C16	27.8 (2)
C2—C3—C4—C5	−1.8 (2)	C20—C15—C16—C17	1.9 (2)
C3—C4—C5—C6	0.5 (2)	C14—C15—C16—C17	−178.35 (14)
C4—C5—C6—O2	−175.45 (14)	C15—C16—C17—C18	−0.2 (3)
C4—C5—C6—C7	2.2 (2)	C16—C17—C18—C19	−1.1 (3)
C3—C2—C7—O3	178.30 (11)	C17—C18—C19—C20	0.7 (3)
C1—C2—C7—O3	−1.65 (18)	C18—C19—C20—C15	1.1 (3)
C3—C2—C7—C6	2.38 (19)	C16—C15—C20—C19	−2.4 (2)
C1—C2—C7—C6	−177.57 (12)	C14—C15—C20—C19	177.90 (15)
O2—C6—C7—O3	−1.64 (18)	C2—C1—N1—O1	179.96 (12)
C5—C6—C7—O3	−179.51 (12)	C5—C6—O2—C8	14.9 (2)
O2—C6—C7—C2	174.22 (12)	C7—C6—O2—C8	−162.82 (15)
C5—C6—C7—C2	−3.7 (2)	C9—C8—O2—C6	156.80 (17)
O3—C10—C11—C14	−93.89 (15)	C2—C7—O3—C10	122.08 (13)
O3—C10—C11—C12	88.35 (13)	C6—C7—O3—C10	−62.01 (15)
C14—C11—C12—O4	174.10 (15)	C11—C10—O3—C7	−149.29 (11)
C10—C11—C12—O4	−8.0 (2)	O4—C12—O5—C13	3.8 (2)
C14—C11—C12—O5	−7.17 (18)	C11—C12—O5—C13	−174.95 (13)
C10—C11—C12—O5	170.71 (11)		

### Hydrogen-bond geometry (Å, º)

Cg2 is the centroid of the C15–C20 ring.

**Table:**

*D*—H···*A*	*D*—H	H···*A*	*D*···*A*	*D*—H···*A*
O1—H1*A*···N1^i^	0.82	2.08	2.8121 (15)	149
C4—H4···O4^ii^	0.93	2.59	3.2379 (18)	127
C20—H20···O1^iii^	0.93	2.59	3.466 (2)	157
C9—H9*B*···*Cg*2^iv^	0.96	2.79	3.616 (2)	145

Symmetry codes: (i) −*x*+2, −*y*, −*z*+2; (ii) −*x*+1, −*y*, −*z*+2; (iii) −*x*+1, −*y*, −*z*+1; (iv) *x*−3/2, −*y*−1/2, *z*−1/2.
